# Analysis of the mRNA export protein ZC3H11A in HCMV infection and pan-cancer

**DOI:** 10.3389/fmicb.2023.1296725

**Published:** 2023-11-15

**Authors:** Jiawen Li, Min Song, Zhen Liu, Fulong Nan, Bin Wang, Dongmeng Qian, Ming Hu

**Affiliations:** ^1^Department of Special Medicine, School of Basic Medicine, Qingdao University, Qingdao, Shandong, China; ^2^Department of Digestive Endoscopy, Qingdao Hospital, University of Health and Rehabilitation Sciences (Qingdao Municipal Hospital), Qingdao, Shandong, China

**Keywords:** ZC3, HCMV, pan-cancer, mRNA export protein, comprehensive analysis

## Abstract

**Background:**

We have previously reported that human cytomegalovirus (HCMV) infection could promote the progression of glioma. Here we discovered a stress-induced nuclear protein ZC3H11A (ZC3) through high-throughput sequencing after HCMV infection, which has been reported recently by our research group in regulating mRNA export under stress conditions. And also, a thorough analysis of ZC3 in pan-cancer and the omics data of ZC3 are yet to be conducted.

**Methods:**

The transcriptomes of glioma cells after HCMV infection were assessed by RNA sequencing. ZC3 mRNA and protein level following HCMV infection were validated and measured by qRT-PCR and Western-blot. The RNA sequencing and protein expression information of ZC3 across pan-cancer were analyzed and visualized by R packages. The localization of ZC3 protein was assessed by IHC images from HPA. The ZC3 proteomics and transcriptomics data in different cancers were extracted through the CPTAC data portal, and comparisons were conducted with a Python script. The genetic alteration, survival prognosis, immune infiltration analysis of ZC3 in pan-cancer were analyzed by cBioPortal, TCGA, and TIMER2 databases. The protein interaction networks were revealed by STRING, GEPIA2 and TCGA.

**Results:**

Genes in mRNA processing pathways were upregulated after HCMV infection and ZC3 expression in mRNA and protein level was validated. We also discovered that the status of ZC3 were generally at high levels in cancers, although varied among different cancer types. ZC3 protein in tumor cells localized to the nuclear whereas in normal cells it was mainly found in cytoplasmic/membranous. However, from ZC3 proteomics and transcriptomics data in some cancer types, the increase in ZC3 protein was not accompanied by a significant elevation in mRNA level. Additionally, our analysis indicated that elevated ZC3 expression was primarily linked to a negative prognosis in majority cancers but still varied depending on the cancer types. Our annotation analysis suggested that ZC3-related proteins are mainly involved in mRNA processing clusters.

**Conclusion:**

We demonstrated that ZC3 significantly impacted by HCMV infection in gliomas. Furthermore, we identified a set of genes exhibiting analogous expression patterns to ZC3H11A in TCGA pan-cancer cohorts, implying a potential functional role for ZC3H11A in mRNA processing. Our study provided valuable insights into the role of a new mRNA export protein ZC3 in HCMV infection and pan-cancer progression. These results lay the foundation for our next research on the regulatory mechanism of ZC3 in virus-infected tumors.

## Introduction

Viral infections have been implicated in the development and progression of various types of tumors through multiple mechanisms. Our previous research have identified that human cytomegalovirus (HCMV) has been implicated as a contributing factor in many cancers (Hu et al., 2017, 2021; Wang et al., 2017; Liang et al., 2019). In the present study, by transcriptomic sequencing and cell line validation, one of the genes that have been shown to be significantly affected by HCMV infection in gliomas is ZC3H11A (ZC3). The discovery of elevated ZC3 expression through HCMV infection of gliomas has provided a unique opportunity to study the expression and function of ZC3 in pan-cancer.

Emerging evidence suggests that ZC3 may have a broader impact on cancer biology beyond gliomas. Our research group recently discovered that the ZC3 gene encodes a stress-induced nuclear protein that is required for efficient reproduction of several nuclear-replicating human viruses (HIV, HSV-1, influenza virus and adenovirus) (Younis et al., 2018). Further, our preliminary results indicate that tumors take advantage of stress-induced ZC3 function(s). A genome-wide association studies (GWAS) in humans also have demonstrated a significant association between the ZC3 gene and susceptibility to breast cancer (Cai et al., 2014). However, there is still a lack of large-scale research to reveal ZC3 expression and function in pan-cancer.

ZC3 is categorized as a CCCH-type zinc finger protein that contains three zinc finger motifs positioned at the N-terminus. Structural prediction programs suggest that a significant portion of the protein, except for the zinc finger and coiled-coil domains, is predominantly intrinsically disordered (Hajikhezri et al., 2020). Zinc finger proteins constitute a vast protein family that possess unique zinc finger (ZnF) domains within their protein sequence. These ZnF domains comprise several ZnF motifs that are short sequences of 30–100 amino acids, coordinating with zinc ions (Zn^2+^). While zinc ions are the preferred metal, zinc finger motifs in ZC3 can bind to other metal ions, such as copper, cobalt, nickel and cadmium, which compete with zinc ions for binding. The functions of ZC3 have remained elusive for a long time. However, two major proteomics studies have shed light on its potential function, revealing that that ZC3 plays a crucial role in the nuclear export of mRNA as it is one of the essential components of the Transcription-Export (TREX) complex (Dufu et al., 2010; Hein et al., 2015). A comprehensive study of the relationship between ZC3 and various cancers, will contribute to our understanding of the regulation of zinc finger proteins in cancer.

In recent years, increasing evidence indicates that zinc finger proteins play important roles in the development and treatment of cancer. Firstly, research has shown that zinc finger proteins play important roles in regulating cancer cell proliferation, apoptosis, and invasion. For example, zinc finger proteins ZEB1 and ZEB2 can promote cancer cell invasion and metastasis by inhibiting the expression of E-cadherin (Ang et al., 2023; Tan et al., 2023). In addition, some zinc finger proteins can inhibit cancer cell apoptosis, such as Bcl-2 family members, which can promote cancer cell survival by inhibiting the mitochondrial apoptotic pathway (Borden, 2020). Secondly, zinc finger proteins are also involved in the regulation of signaling pathways in cancer development. For example, zinc finger protein Nrf2 can promote cancer cell metastasis and proliferation by regulating the oxidative stress response, while GATA3 can inhibit cancer cell angiogenesis and invasion (Wu et al., 2023).

Several cancers have been linked to abnormal ZC3 expression or protein interactions. For instance, ZC3 expression levels are considerably higher in breast cancer tissues than in normal tissues (Cai et al., 2014; Tabl et al., 2018). Moreover, a significant upregulation of ZC3 has been observed in KRAS-mutant lung adenocarcinomas (Grzeskowiak et al., 2018), where KRAS mutations are frequently observed molecular alterations in non-small cell lung cancers and serve as a predictor of poor prognosis (Buscail et al., 2020). In addition, ZC3 has been reported to interact with a mutant form of the nuclear matrix protein Matrin-3, which is present in amyotrophic lateral sclerosis (ALS) patients. This study demonstrated that ALS-associated mutations increase the co-localization of Matrin-3 with components of the TREX complex, possibly explaining the nuclear mRNA export defects observed in ALS patients (Boehringer et al., 2017). The export of mRNAs is a process that responds to a range of cellular stimuli and stressors. Primary human cancer specimens have exhibited abnormal RNA export. These exported RNAs encode factors that play crucial roles in almost all aspects of malignancy (Borden, 2020). Therefore, the function of ZC3 in regulating mRNA nuclear export may potentially have an impact on tumors.

Herein, in HCMV infected glioma, we identified an abnormal expression of ZC3, an mRNA nuclear export gene, by transcriptome sequencing and subsequent qRT-PCR and western-blot verification in cell lines. We also established a systematic analysis to investigate the association of ZC3 in primary tumors using the TCGA pan-cancer data, and compared the ZC3 proteomics and transcriptomics data for each type of pan-cancer patients from the National Cancer Institute's Clinical Proteomic Tumor Analysis Consortium (CPTAC) dataset. The results of this study offered insights into the panoramic view of ZC3 in cancer, including its underlying causes and potential consequences. It also deepened the understanding of the relationship between RNA-binding proteins and tumors. These research findings are significant for targeting ZC3 in cancer treatment as they identify vulnerable cancer types and potential biomarkers that can be further investigated for therapeutic purposes.

## Materials and methods

### Cell culture and virus infection

The U87 glioma cell line was acquired from the Shanghai Cell Bank of the Chinese Academy of Sciences (Shanghai, China) and was maintained in MEM at 37°C under 5% CO_2_. The HCMV strain AD169 and virus infection procedures were conducted as described previously (Hu et al., 2021). Cells were grown in a serum-free medium and exposed to HCMV at the multiplicity of infection (MOI) of 1. After a 2-h incubation at 37°C, the medium was replaced with a complete one. Simulated infections were carried out separately and concurrently.

### RNA-seq and bioinformatics

U87 glioblastoma cells were infected with human cytomegalovirus (HCMV) at a MOI of 1. The cells were then incubated for a period of 2 days. Total RNA was extracted from the cells using TRIzol, following the protocol provided by the manufacturer. To prepare the mRNA libraries, the Illumina TruSeq strand-specific mRNA-seq library preparation kit was used. The libraries were subsequently sequenced on the NextSeq 500 platform, generating paired-end reads. The mRNA sequencing data have been deposited in the European Nucleotide Archive (ENA) under the Accession Number PRJEB30943. All subsequent analyses were performed on clean data, as described in detail in the Results section. We computed the significance of genes remained after filtering with FDR < 0.05 as a threshold by DESeq2 R package. All data analysis was conducted on a server running the CentOS7 operating system, provided by Qingdao University.

### Reverse transcription and quantitative real-time PCR

The extraction of RNA was conducted utilizing Trizol reagent (Invitrogen) in accordance with the guidelines provided by the manufacturer. For the synthesis of first-strand cDNA, the first strand cDNA synthesis kit (MBI Fermentas, St Leon Roth, Germany) was employed. The quantification of reverse transcription-polymerase chain reaction (qRT-PCR) was carried out using the Bio-Rad iCycler IQ5 system in conjunction with SYBR Green Master mix. The expression levels of mRNA in each sample were adjusted to the internal control of endogenous origin. The forward and reverse primers for ZC3 detection were 5′-AAGGAAGGACTTACCCATTTTGATATT-3′ and 5′-TGGGTCAGATTTCCCTATGAGAA-3′, respectively. The forward and reverse primers for HCMV UL122 detection were 5′-TGACCGAGGATTGCAACGA-3′ and 5′-CGGCATGATTGACAGCCTG-3′, respectively. The forward and reverse primers 5′-TGGAACGGTGAAGGTGACAG-3′ and 5′-GGCTTTTAGGATGGCAAGGG-3′ were used to detect β-actin as the internal control.

### Western blot analysis

Cell harvesting and protein extraction were carried out using RIPA with PMSF. SDS-polyacrylamide gel electrophoresis was employed to separate the extracted proteins. Following separation, the proteins were transferred onto a PVDF membrane (Merck Millipore, USA) and blocked using 5% skim milk. The blots were then probed with the ZC3 primary antibody (ab99930, Abcam, USA) at a dilution of 1:1,000 and the HCMV IE antibody (ab53495, Abcam, USA) at a dilution of 1:1,000. The β-actin antibody (bs-10966R, Bioss, China) was used as an internal reference at a dilution of 1:2,000. Subsequently, the secondary antibodies and ECL western blotting detection were performed following established protocols (Hu et al., 2021).

### An analysis of ZC3 expression across pan-cancer

We obtained the ZC3 (ENSG00000058673) RNAseq data for TCGA gene expression (level3, HTSeq-FPKM). The fregments per kilobase per million (FPKM) were subsequently transformed and normalized to transcripts per million (TPM). All cancer types were identified using their TCGA four-letter abbreviation (refer to abbreviations). The R language for statistical computing (version 3.6.3) was utilized to conduct all statistical analyses. The ggplot2 package was used for data visualization. To achieve a normal distribution, the normalized counts were log_2_ transformed prior to conducting any statistical analyses. Box or violin plots were created using expression data that had been transformed with log_2_ (TPM + 1). The normalized cancer and adjacent normal data for visualization has been uploaded as Supplementary material as median with the first and third quartile (Q1, Q3).

To analyze protein expression in primary tumor and normal tissues from the CPTAC dataset, we utilized the UALCAN portal (http://ualcan.path.uab.edu/analysis-prot.html), an interactive web resource. Our analysis focused on examining total protein and phosphorylation levels of ZC3. *Z*-values were used to represent standard deviations from the median across samples for the specific cancer type. Log_2_ Spectral count ratio values from CPTAC were normalized within each sample profile and across samples. The normalized statistical significance data between cancer and normal sample for visualization has been uploaded as Supplementary material include maximum, minimum, median and the first and third quartile (Q1, Q3).

To assess localization in ZC3 expression on a protein level, we obtained immunohistochemistry (IHC) images of ZC3 protein expression from both normal and tumor tissues. We accessed these images from the Human Protein Atlas website (http://www.proteinatlas.org/) for analysis.

For the proteomics vs. transcriptomics comparison, raw datasets in the field of proteomics and transcriptomics are accessible through the CPTAC data portal in a Python programming environment. We imported the Omics Data package and loaded the data with a standard Python import statement. Then we extracted the data and compared the proteomics and transcriptomics data from different type of cancer patients for the ZC3 gene. Finally, we plotted the transcriptomics data against the proteomics data with Seaborn library. A Python script for processing colon cancer has been uploaded in the Supplementary material.

### Genetic alteration analysis

For genetic alteration analysis, the cBioPortal tool (https://www.cbioportal.org) was used to gather information on the genetic alterations of ZC3 in all TCGA tumors. The “TCGA Pan Cancer Atlas Studies” option in the “Quick select” section was selected, and the genetic alteration characteristics of the ZC3 gene were queried. The “Cancer Types Summary” module provided results on mutation type, alteration frequency, and copy number alteration (CNA) across all TCGA tumors. The “Mutations” module was consulted for mutated site information, protein structure schematic diagrams, or 3D structures. The “comparison” module was used to gather data on overall, disease-free, progression-free, and disease-free survival differences among TCGA cancer cases with or without ZC3 genetic alterations. Kaplan–Meier plots were generated, and log-rank *p*-values were computed.

### Survival prognosis analysis

The “Survival Map” module of GEPIA2 was employed to obtain the OS (Overall survival) and DFS (Disease-free survival) significance map data of ZC3 in all TCGA tumors. We utilized expression thresholds of cutoff-high (50%) and cutoff-low (50%) to divide cohorts into high-expression and low-expression groups. The log-rank test was conducted to evaluate the hypothesis, and survival plots were generated using the “Survival Analysis” module of GEPIA2.

### Immune infiltration analysis

For immune infiltration analysis, the “Immune-Gene” module of the TIMER2 web server was utilized to evaluate the correlation between ZC3 expression and immune infiltrates across all TCGA tumors, with a focus on CD8^+^ T-cells and cancer-associated fibroblasts. Immune infiltration was estimated using algorithms such as MCPCOUNTER, CIBERSORT, TIMER, CIBERSORT-ABS, QUANTISEQ, XCELL, and EPIC. The purity-adjusted Spearman's rank correlation test provided *p*-values and partial correlation (cor) values. Data was presented through scatter plots and heatmaps.

### ZC3-related proteins enrichment analysis

To analyze the protein-protein interaction network, we used the STRING website (https://string-db.org/) and set the following main parameters for analysis: “Low confidence (0.150)” for minimum required interaction score, “evidence” for meaning of network edges, “no more than 50 interactors” in 1st shell for max number of interactors to show, and “experiments” for active interaction sources.

We utilized GEPIA2 to collect the leading 100 ZC3-linked genes from the comprehensive TCGA tumor and normal tissue datasets. Following this, we performed a gene-gene Pearson correlation analysis between ZC3 and the chosen genes. Furthermore, we generated a heatmap to visually represent the expression profile of the selected genes alongside the purity-adjusted Spearman's rank correlation test results for partial correlation (cor) and *p*-value.

To conduct Kyoto Encyclopedia of Genes and Genomes (KEGG) pathway analysis, two datasets were merged and filtered. To visualize the enriched pathways, we utilized the “tidyr” and “ggplot2” R packages. We performed this analysis using R-3.6.3, 64-bit, which was deemed appropriate for our study. A two-tailed *p*-value of < 0.05 was deemed statistically significant for this analysis.

### Statistical analysis

Statistical comparisons were performed using unpaired student *t*-test or one-way analysis of variance (ANOVA). Data normality was assessed using the Shapiro-Wilcoxon normality test and D'Agostino & Pearson test. The data were presented as means ± standard deviation (SD), and a *p*-value of < 0.05 was considered statistically significant. GraphPad Prism software version 8 (La Jolla, CA) was used for data analysis.

## Results

### HCMV infection upregulates ZC3H11A expression in glioma cells

**Figure 1:**
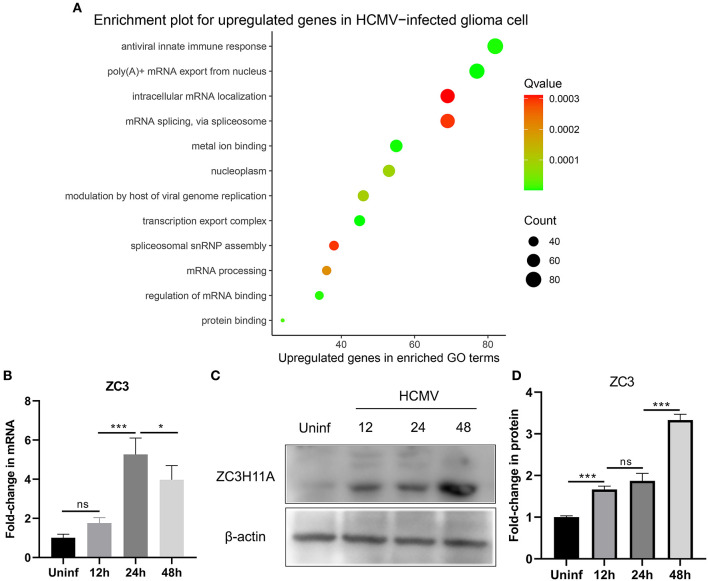
Effect of HCMV infection on the transcriptome and expression of ZC3 in glioma cells. **(A)** The enriched functional pathways for upregulated genes after HCMV infection. **(B)** Quantitative PCR analysis of ZC3H11A mRNA expression levels after HCMV infection. **(C, D)** Immunoblot detection of ZC3H11A protein levels during HCMV infection. *p < 0.05, ***p < 0.0001.

Our recent study has provided evidence that infection with human cytomegalovirus (HCMV) can induce specific reactions within cells and contribute to the advancement of glioma. These reactions are characterized by an elevation in the growth and proliferation of glioma cells, along with a heightened resistance to apoptosis. In order to gain a comprehensive understanding of the cellular responses involved, we utilized a genome-wide transcriptome analysis. In this study, we infected glioma cells with HCMV and conducted a transcriptome analysis comparing infected cells to mock-infected cells at 2 days post-infection (dpi). This time point was chosen based on our previous demonstration that the host response is already induced and reaches its peak at this time (Dufu et al., 2010). By focusing on the genes that were most significantly upregulated in HCMV-infected glioma cells, we observed a substantial increase in the expression of stress-induced genes, as well as mRNA processing pathways. In our study, we made an interesting discovery regarding the gene ZC3, which showed minimal expression in cells not infected with HCMV. The Gene ontology (GO) pathway analysis was performed using ClusterProfiler and org.Hs.eg.db R package and enriched functional groups following HCMV infection are depicted in bubble plot (Figure 1A). This finding is particularly noteworthy because HCMV infection is known to cause a global decrease in the levels of host cell mRNA (Isler et al., 2005). Previous research on ZC3 has primarily focused on its function in adenovirus, and its role in HCMV infection has not been explored before.

In order to validate these findings, we utilized quantitative real-time reverse transcription-PCR (RT-PCR) to analyze the RNA extracted from both infected and mock-infected glioma cells. The results of this analysis provided further evidence supporting the transcriptome analysis that revealed the upregulation of ZC3 expression in glioma cells following HCMV infection (Figure 1B). Interestingly, HCMV infection also resulted in a significant increase in the ZC3H11A protein at late time points of infection (Figures 1C, D). Since HCMV induces a widespread inhibition of the host's protein production, while simultaneously facilitating the translation of its own viral mRNAs. The results suggest that ZC3 is significantly impacted by HCMV infection in gliomas.

### Analysis of ZC3 mRNA and protein expression in pan-cancer

**Figure 2:**
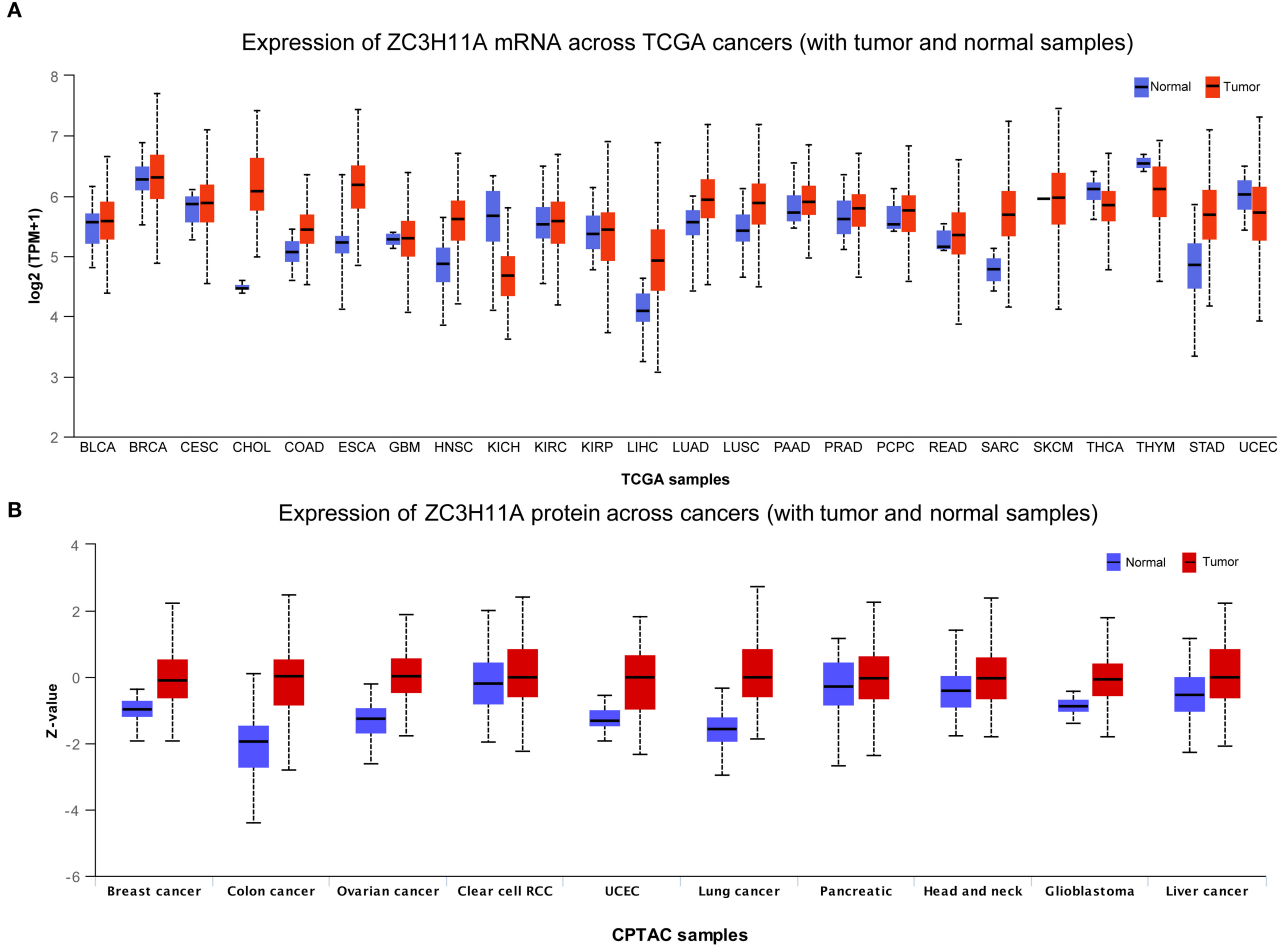
The mRNA and protein expression of ZC3 in human tumors. **(A)** The mRNA expression [log_2_ (TPM + 1)] of ZC3 in TCGA tumors vs. adjacent tissues as visualized by ggplot2. The sample lines indicate the medians and quartiles. **(B)** The protein level of ZC3 in normal tissue and primary tumors. The analysis of protein data was performed using CPTAC.

In order to verify our hypothesis of ZC3, in this section we aimed to provide an in-depth evaluation of ZC3 expression across TCGA normal tissues and TCGA cancer types. UCSC TOIL was used to correct for batch effects and merge samples. RNAseq data in transcripts per million reads (TPM) format was subjected to analysis and comparison after log_2_ transformation. Twenty-four sets of comparable tumors and normal tissues were eventually generated (Supplementary Table S1). In general, ZC3 mRNA exhibited a distinct elevated expression pattern in pan-cancer compared to the corresponding normal tissue. As determined by *T*-test, ZC3 mRNA expression was significantly higher in 11 types (11/24, *p* < 0.01) of tumors compared to normal tissues. In contrast, ZC3 mRNA expression was lower in tumor tissues than in normal in 3 (3/24, *p* < 0.01) types tumors (Figure 2A, Supplementary Table S2). Comparing across cancer types, ZC3 expression varied broadly suggesting that high ZC3-expressing cancers may be driven by unique genetic features (Figure 2A). Interestingly, among the high ZC3 expression cancer types, we noticed a more significant differences in gastrointestinal neoplasms and digestive system cancers (CHOL, ESCA, LIHC and STAD). Moreover, according to the interquartile range (IQR), ZC3 mRNA expression was more widespread in some types of cancer than others; e.g., bile duct cancer (CHOL) had a wide spread while pancreatic cancer (PAAD) had a narrow spread.

Next, a comparison of ZC3 protein expression across the TCGA was also conducted. Although the expression of ZC3 protein varied across tumor types, unlike ZC3 mRNA expression, ZC3 protein was much higher in tumor tissue than normal tissue in almost all cancers (Figure 2B, Supplementary Table S3). ZC3 protein followed the same expression patterns throughout most tumor types.

**Figure 3:**
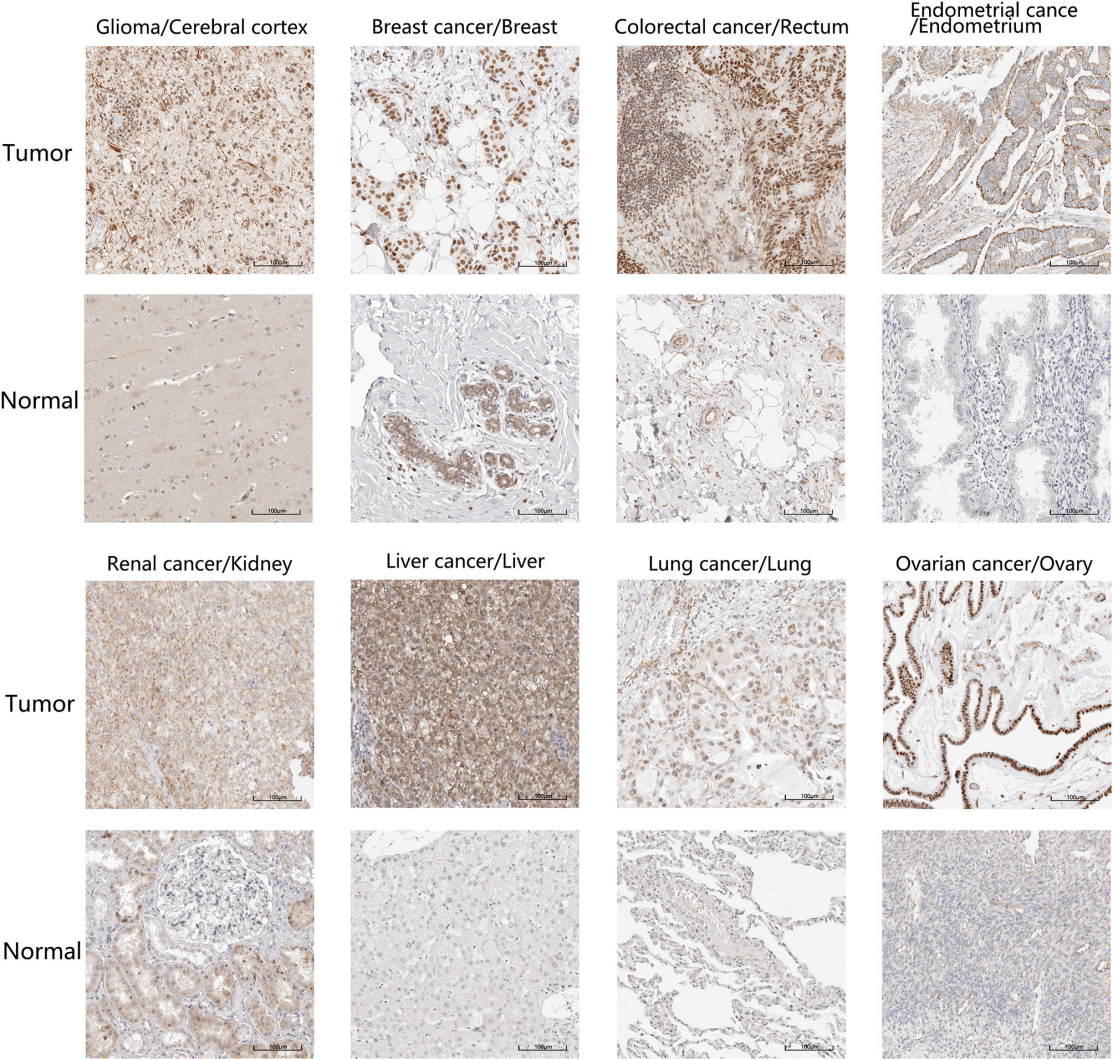
The expression and intracellular localization of ZC3 protein. The expression and intracellular localization of ZC3 protein that assessed by immunostaining in tumors and the corresponding normal tissues from HPA.

Additionally, we localized the ZC3 protein expression using tumor histological staining obtained from HPA. As expected, ZC3 protein staining was positive in all selected tumor tissue showing moderate and/or strong staining intensity. In contrast, ZC3 was mainly detected in a salt-and-pepper like pattern with low staining or not detected in corresponding normal tissue (Figure 3). The neuronal cells in cerebral cortex and tubular cells in kidney were two exceptions, because these cells showed moderate intensity with around 75% quantity for ZC3 staining. The cervical adenocarcinoma cells displayed continuous positive for ZC3 while ovarian stroma cells displayed discontinuous staining. Interestingly, ZC3 protein in tumor cells localized to the nuclear whereas in normal cells it was mainly found in cytoplasmic/membranous (Figure 3).

**Figure 4:**
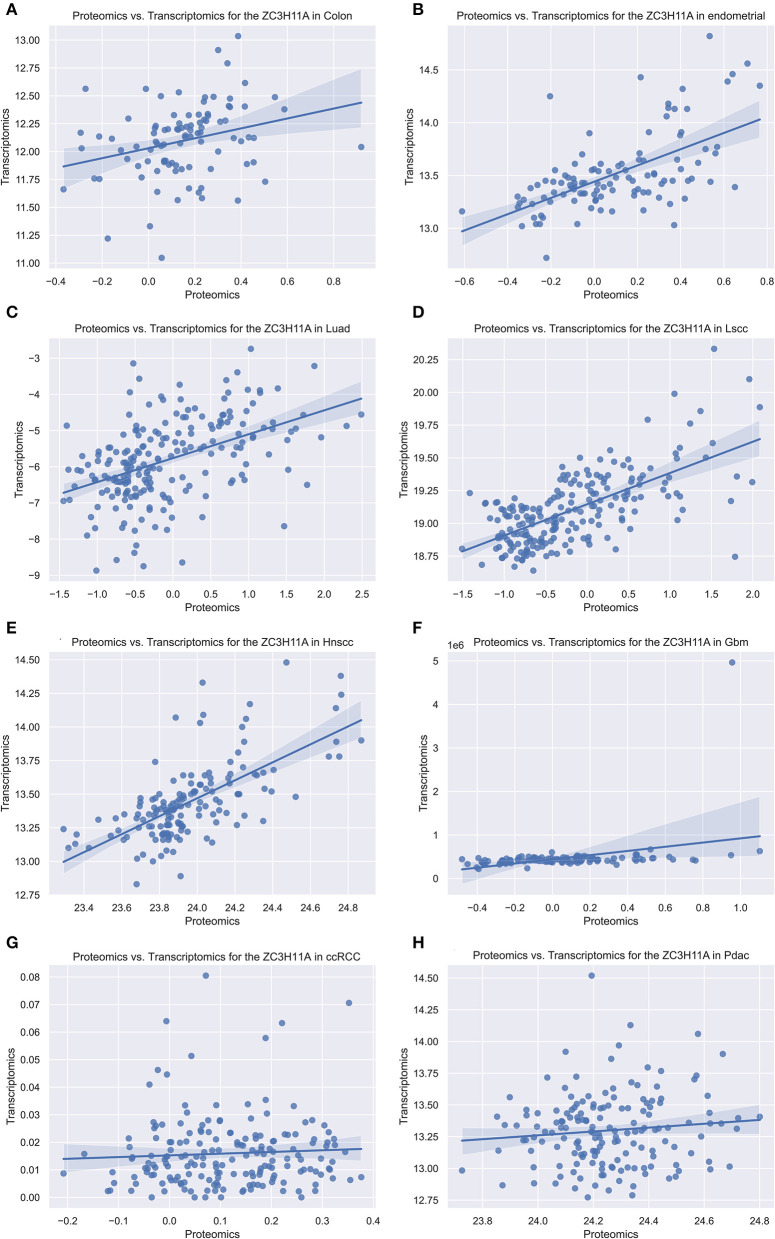
The scatterplot comparing transcriptomics and proteomics of ZC3 in pan-cancer with CPTAC dataset. The proteomics and transcriptomics data from the pan-cancer patients were compared using the National Cancer Institute's CPTAC program as a Python package. **(A–H)** Represent different cancer types and the abbreviated names are listed on each title.

As our group previously demonstrated that ZC3 protein levels were significantly elevated under stress condition, while mRNA levels were not elevated apparently. In order to determine this correlation in tumor, we compared the ZC3 proteomics and transcriptomics data for each type of pan-cancer patients with Python code from the National Cancer Institute's Clinical Proteomic Tumor Analysis Consortium (CPTAC) dataset. The results showed that ZC3 Protein and mRNA changes were highly correlated for most cancer types (5/8, Figures 4A–E). However, in glioblastoma (GBM), kidney clear cell carcinoma (ccRCC) and pancreatic ductal adenocarcinoma (PDAC), the increase in ZC3 protein was not accompanied by a significant elevation in mRNA level (Figures 4F–H). This observation indicates that ZC3 protein may be more dynamically involved in post-translational levels in certain cancer types.

### Analysis of ZC3 phosphorylation in certain cancer types

**Figure 5:**
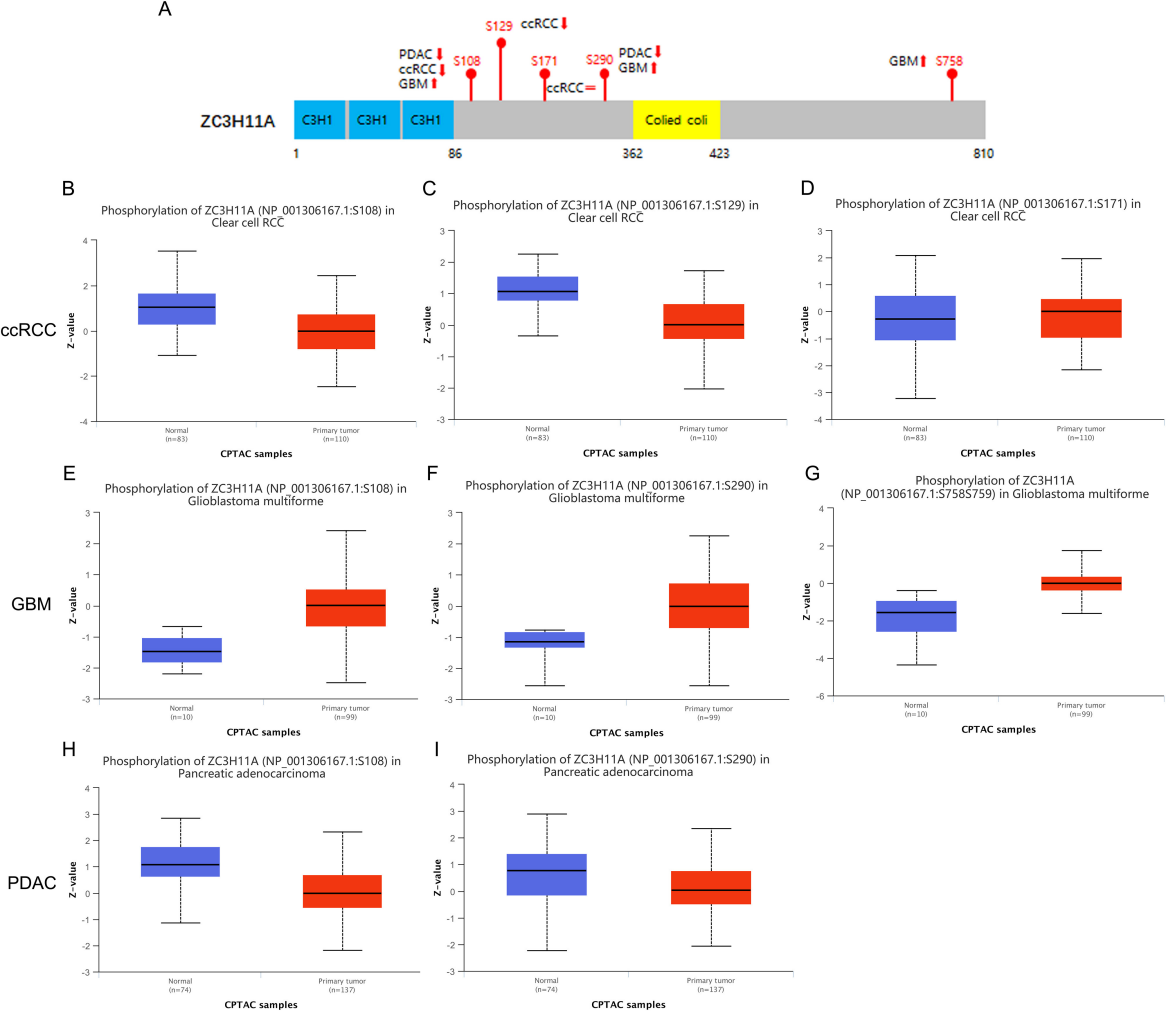
Tumor-associated protein phosphorylation of ZC3. **(A)** A schematic diagram of ZC3 phosphoprotein sites and summarized phosphorylation levels in primary tumors. **(B–D)** Box plot representation of ZC3 phosphorylation levels at different sites in ccRCC. **(E–G)** Box plot representation of ZC3 phosphorylation levels at different sites in GBM. **(H, I)** Box plot representation of ZC3 phosphorylation levels at different sites in PDAC. *Z*-values represent standard deviations from the median across samples for the given cancer type.

Since ZC3 may undergo various posttranslational modifications, including extensive regulation via phosphorylation, in three cancer types (ccRCC, GBM, and PDAC) with low correlation between ZC3 mRNA expression and protein abundance. We next compared the phosphorylation of ZC3 between primary tumors and normal tissues. Based on mass spectrometry analysis and phospho-proteomics data, ZC3 unique phosphorylation sites have been identified in cancers (Beausoleil et al., 2006; Dephoure et al., 2008; Zhou et al., 2013). As summarized in Figure 5A, the phosphorylation level at S108 (*p* = 6.796E-10) and S290 (*p* = 6.507E-04) was both reduced in PDAC, while the phosphorylation level at these same sites was significantly increased in GBM compared to normal tissues (*p* = 6.888E-07 and *p* = 1.85E-03; Figures 5E–I). In ccRCC the phosphorylation level was reduced at S108 (*p* = 4.347E-11) and S129 (*p* = 3.991E-10) and was no significant differences at S171 (*p* = 0.703; Figures 5B–D). The phosphorylation level of ZC3 protein can generally be categorized as being tumor specific compared to normal tissues.

### Genetic alteration analysis of ZC3 in pan-cancer

**Figure 6:**
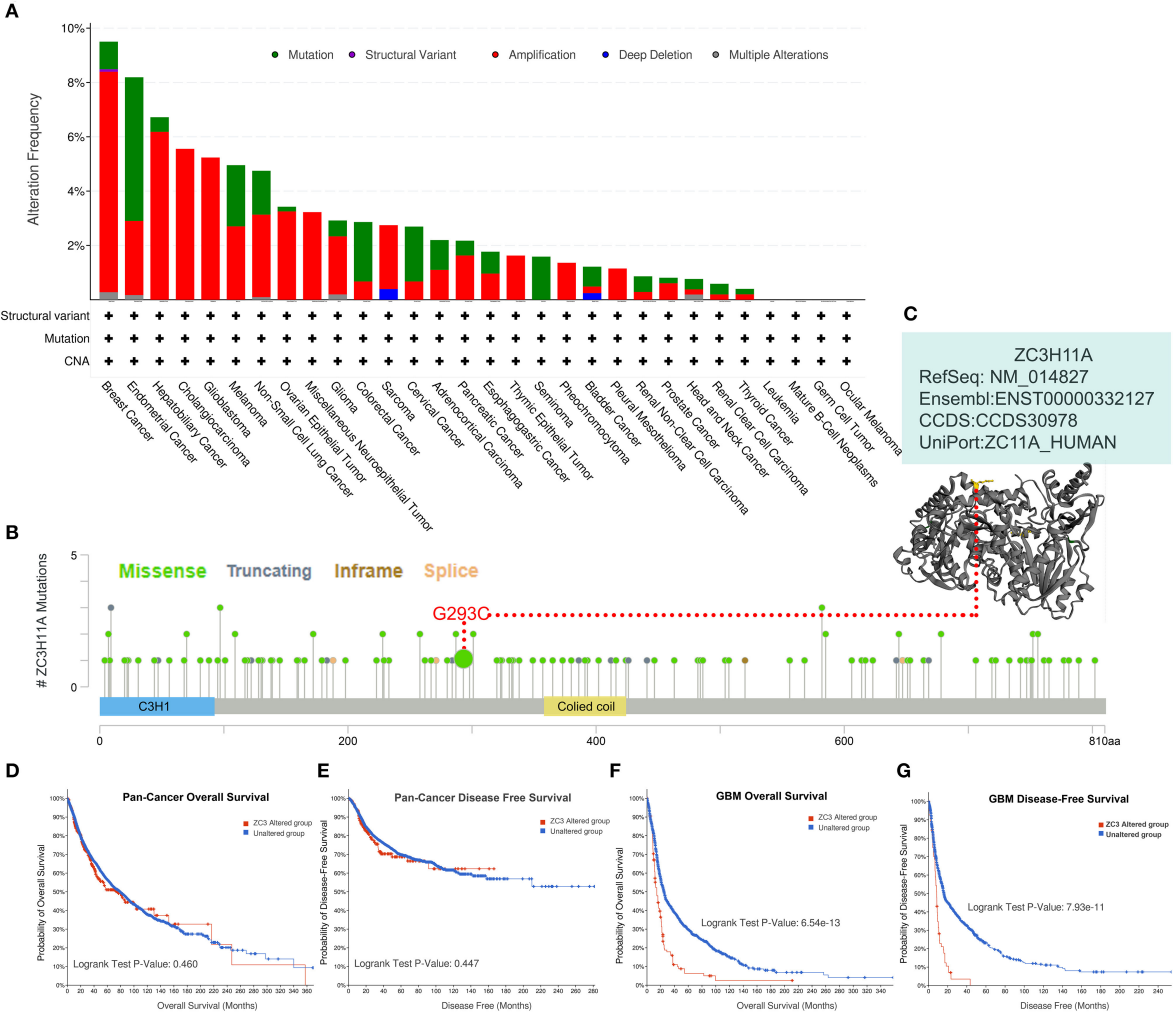
Genetic alteration status of ZC3 in pan-cancer. **(A)** The alteration frequency and mutation type of ZC3 in different cancer types. **(B)** The amino acid mutation sites of ZC3 protein and **(C)** a mutated crystal structure of aminotransferase in 293aa. **(D, E)** The correlation between ZC3 alteration status and pan-cancer overall survival or disease-free survival. **(F, G)** The correlation between ZC3 alteration status and GBM overall survival or disease-free survival.

Genetic alterations are responsible for the development of human cancers. We proceeded to investigate the genetic alterations in ZC3 in human tumor samples. Based on findings from TCGA Pan-cancer Studies, the alteration frequency of ZC3 (>9%) was observed to be highest in breast cancer, with “Amplification” identified as the primary type. Endometrial Cancer had the second highest alteration frequency (>9%) of ZC3 with the incidence of “Mutation” as the primary type (Figure 6A). Figure 6A also showed the alteration frequency across other types of cancer. The main features of the mutations type were “Mutation” and “Amplification.” We further presented additional mutations and their location within ZC3 (Figure 6B). It appeared that there was no main type of genetic alteration and the genetic alteration were rather sporadic with some locating in ZnF motifs (C3H1) and colied coil motifs. Figure 6C shows the visualized mutated amino acids G293C within a crystal structure of ZC3.

Next, a systematical investigation was conducted to determine the correlation between clinical survival prognosis and certain genetic alterations of ZC3 in various types of tumors. Based on all combined TCGA PanCancer Atlas, altered ZC3 (red line) was not associated with overall survival (OS, *p* = 0.460) or disease-free survival (DFS, *p* = 0.447; Figures 6D, E). Conversely, the OS and DFS in patients with ZC3 alterations was much significant in GBM cases (Figures 6F, G). Together, these findings suggest ZC3 genetic alterations may play a role in the development of some special types of tumors.

### Association of ZC3 expression with patient survival

**Figure 7:**
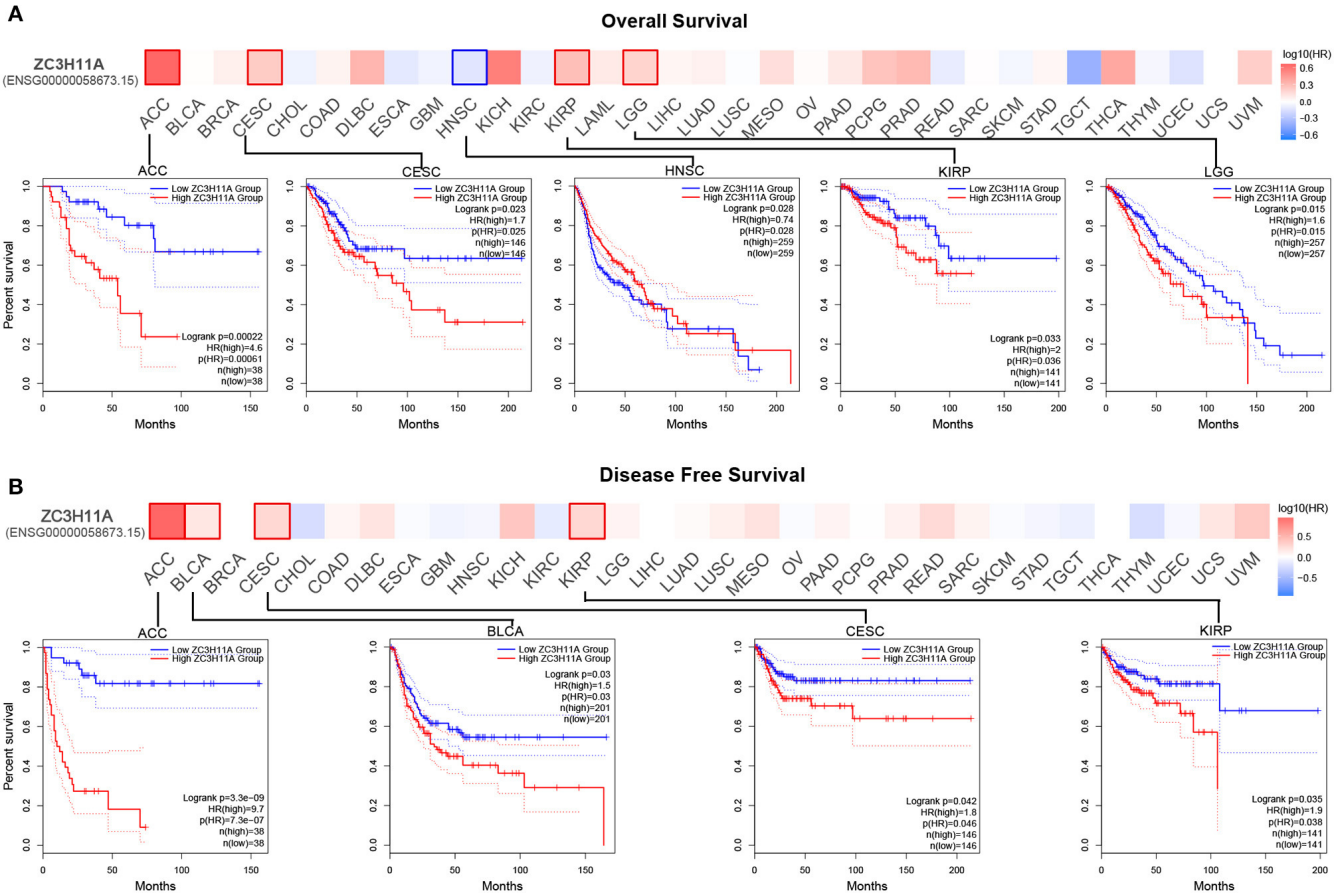
The correlation between ZC3 expression and the patient survival rate across different types of cancer. **(A)** The correlation between ZC3 expression and patient overall survival by Gepia2 tool analysis. **(B)** The correlation between ZC3 expression and patient disease-free survival by Gepia2 tool analysis.

We then sought to investigate the correlation between ZC3 expression and prognosis in pan-cancer. Based on ZC3 expression level, cancer cases were divided into two groups: low ZC3 expression and high ZC3 expression. Our analysis of the GEPIA2 TCGA data set revealed the correlation between ZC3 expression and patient OS or DFS varied depending on the cancer type tested as shown in Figure 7. More specifically, increased expression of ZC3 was primary associated with overall survival (OS) disadvantage and predicted poor prognosis of patients with, Cervical Squamous and Endocervical Adenocarcinoma (CESC), Kidney Papillary Cell Carcinoma (KIRP), Adrenocortical Carcinoma (ACC) and Lower Grade Glioma (LGG; Figure 7A). In contrast, increased ZC3 expression was associated with increased survival advantage in Head and Neck Squamous Cell Carcinoma (HNSC). While increased ZC3 expression only predicted poor prognosis with disease free survival (DFS) of patients with ACC, Bladder Urothelial Carcinoma (BLCA), CESC and KIRP (Figure 7B).

In order to confirm these results, we also used Starbase and Kaplan-Meier plotter tool to analyze the survival data. The results were similar to those with GEPIA2, that a primary correlation between high ZC3 expression and OS disadvantage was noted in Starbase, where ZC3 predicted poor prognosis of ACC, KIRP, LGG and moreover Kidney Chromophobe (KICH; Supplementary Figure S1A). The Kaplan-Meier plotter tool showed an increased survival disadvantage with ZC3 high expression in CESC, KIRP, and Liver Hepatocellular Carcinoma (LIHC) but predicted better survival for patients with HNSC and moreover Esophageal Carcinoma (ESCA; Supplementary Figure S1B and Supplementary Table S4). The results showed that ZC3 high expression was primary associated with poor prognosis in majority cancers but still varied depending on the cancer types.

### Association between ZC3 expression and immune infiltration

**Figure 8:**
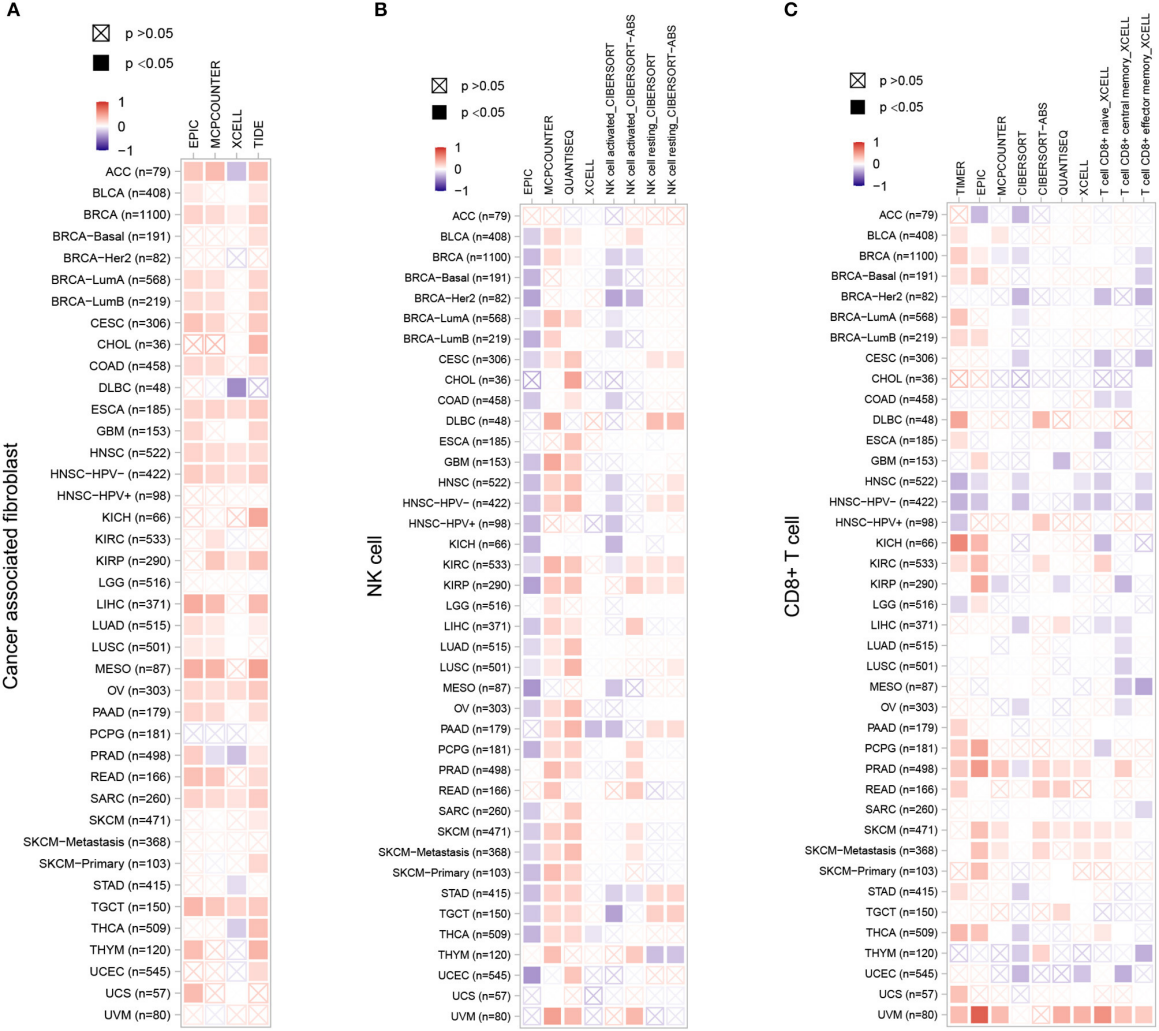
The correlation between ZC3 expression levels and infiltration of immune cells. For the correlative analysis of the ZC3 gene and CAFs **(A)**, NK cells **(B)**, and CD8^+^ T cells **(C)** across all tumors in TCGA. The following algorithms were utilized: EPIC, TIDE, quanTIseq, CIBERSORT, TIMER, XCELL, and MCPCOUNTER. A positive correlation (0–1) is indicated in red, while a negative correlation (−1 to 0) is depicted in blue. A correlation with a *p*-value < 0.05 is considered statistically significant. Correlation values that are statistically non-significant are marked with a cross.

Stromal cells which surrounding cancer cells caner support cancer cell malignancy and are essential for tumor progression. Among the stromal cells, cancer-associated fibroblasts (CAFs) are the most abundant. Therefore, we chose CAFs, NK cells and CD8^+^ T cells to investigate the relationship between ZC3 expression and the infiltration level of pan-cancer types. The correlation was calculated by a variety of algorithm for the quantification of immune cells such as EPIC, XCELL, quanTIseq, TIMER, and so forth (Figure 8). As shown, a significant positive correlation between ZC3 gene expression and the presence of cancer-associated fibroblast (CAF) cells (Figure 8A) was observed in almost all types of cancer, suggesting that high ZC3 gene expression is linked to an active stromal component. For NK cells (Figure 8B), compared to EPIC and CIBERSORT methods, quanTIseq robustly obtained positive correlations across all cancer types. In contrast, MCPcounter and quanTIseq had a significant positive correlation. Because tumor purity and immune infiltration are inevitably negatively correlated for techniques like EPIC, which provide cell fractions referred to as total cells. In the minor cluster consisting of HNSC, THYM and UCEC, a significant negative correlation was observed between ZC3 expression and CD8^+^ T cells, but weak or no correlation in the other clusters as LUAD, LUSC, MESO, and OV (Figure 8C).

It is worth noting, in nearly all cancer types, innate immune system and stromal component exhibited a positive correlation with ZC3 expression, providing further evidence that samples displaying a hot Targeting Tumor Microenvironment (TME) may be linked to increased ZC3 gene expression.

### The main function of ZC3-related proteins was annotated to mRNA processing cluster

**Figure 9:**
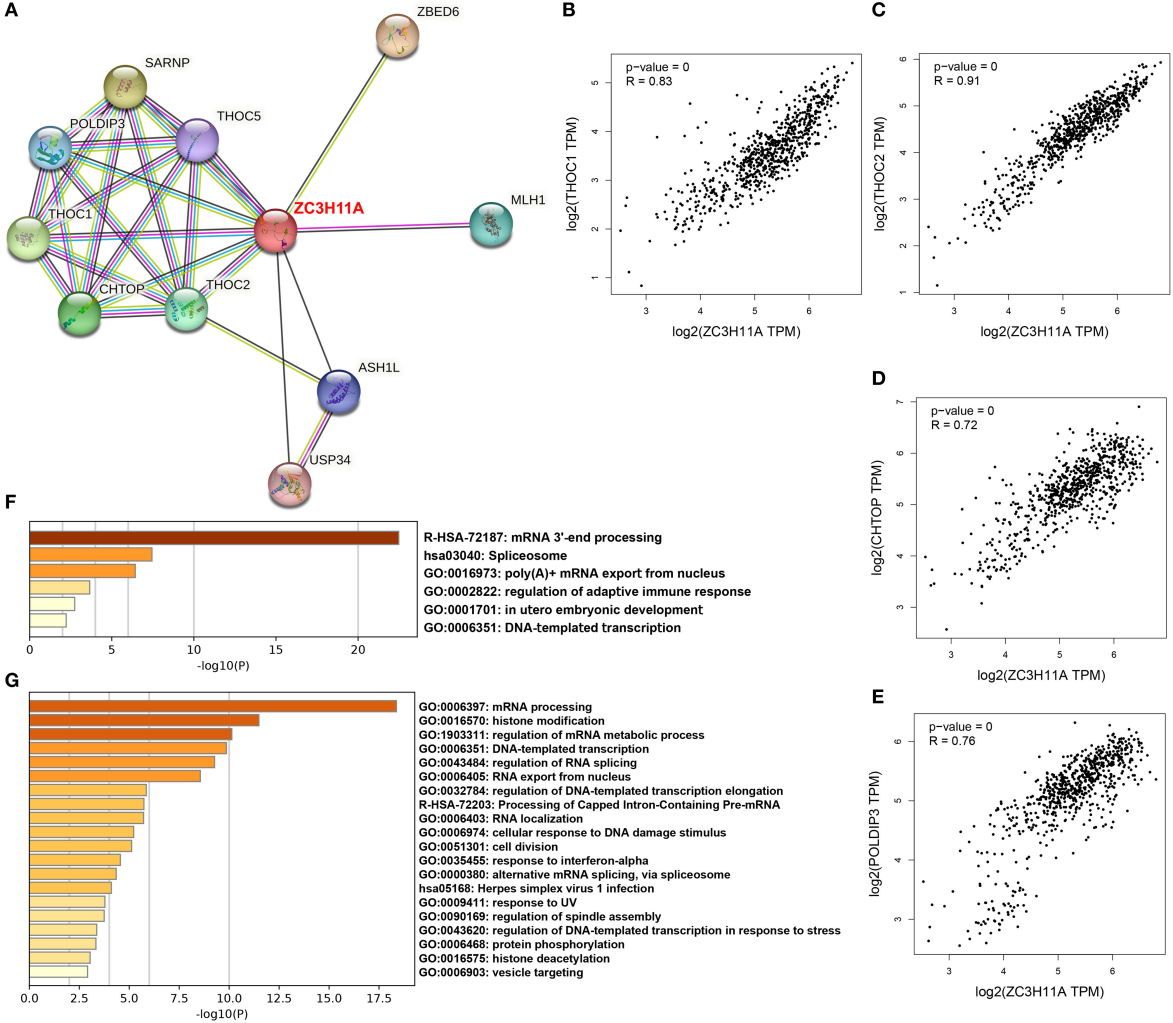
ZC3-related proteins and functional clustering analysis. **(A)** An experimentally determined protein-protein interaction network of ZC3 based on STRING database. The identified proteins are indicated by the colored nodes. **(B–E)** The expression correlation in pan-cancers between ZC3 and ZC3-interacted proteins. **(F)** The GO functional annotation clustering of ZC3-interacted proteins. **(G)** The GO functional annotation clustering of proteins which have a similar expression pattern with ZC3 in pan-cancers.

To date, only few studies have addressed the ZC3 interactions. We explored large-scale proteomics and STRING database to discover protein–protein interactions and potential functional clustering. By mining the reported proteomics studies, we identified 14 confirmed interactions, and most of these are the Transcription and EXport (TREX) complex (Prigge et al., 2009; Dufu et al., 2010; Hein et al., 2015). The STRING analysis result revealed more interactions which are also associated with TREX complex or mRNA processing (Figure 9A). We selected the shared proteins between the two sources and computed the expression correlation analysis with ZC3 in pan-cancer types. We observed a pan-cancer positive correlation between ZC3 and many TREX complex proteins (Figures 9B–E). We also picked up the 14 interacted proteins from reported proteomics and the experimentally determined interactions from STRING to perform gene ontology (GO) functional annotation clustering analysis. The result revealed enriched terms for mRNA processing, spliceosome and mRNA export (Figure 9F).

In addition to direct interactions, we further identified a list of partners which have a similar expression pattern with ZC3 under TCGA pan-cancer types. Consistent with our prediction, the functional clustering of these genes robustly enriched in mRNA processing or splicing terms (Figure 9G). Interesting, GO functional clustering results also showed clusters of herpes virus infection and stress response (Figure 9G). The results of this section strongly imply a functional role for ZC3 in mRNA processing.

## Discussion

The complex interplay between viral infections and oncogenesis has been a subject of considerable interest in recent years. Employing a global transcriptome analysis of infected human glioma cells, we found that the ZC3 gene was one of the most profoundly upregulated mRNA export genes following HCMV infection compared to uninfected controls. Importantly, at 48 h post infection, the elevation of ZC3 protein was more pronounced than the mRNA level. This observation suggests that HCMV infection plays a crucial role in the dysregulation of ZC3 in gliomas, potentially contributing to tumor development and progression.

There are several reasons why we believe that the ZC3 may be important for cancer biology. Firstly, ZC3 is a stress-induced protein and may therefore be essential during tumor growth, for instance due to hypoxia. Secondly, ZC3 is a member of the zinc finger CCCH-type containing protein family, which recently has been demonstrated to play a role in RNA processing and splicing (Younis et al., 2018; Hajikhezri et al., 2020). The research on RNA regulatory proteins in tumors is by far not fully explicated (Hu et al., 2022). This study provides a comprehensive analysis of the relationship between ZC3 and pan-cancer, with a focus on mRNA and protein expression, proteomics and transcriptomics correlation, genetic alterations, patient survival, post-translational modification, and immune infiltration.

Firstly, our study showed a significant upregulation of ZC3 mRNA and protein expression in most cancer types compared to normal tissues, indicating that the over-expression of ZC3 is a common phenotype in cancer, although the pattern and extent of this loss may differ depending on the cancer type. We identified that ZC3 mRNA expression was more widespread in some types of cancer than others. This could be because some cancers have multiple clearly defined subtypes, increasing their genetic diversity. This indicates that ZC3 may be playing diverse roles in different cancer types, possibly due to variations in their molecular characteristics. Interestingly, ZC3 protein in tumor cells localized to the nuclear whereas in normal cells it was mainly found in cytoplasmic/membranous. This result is consistent with the RNA processing function of ZC3 and suggests that tumor cells may require more ZC3 to play a specific role in RNA processing within the nucleus, including transcription and RNA export. The data on subcellular localization serve as a valuable asset in defining the protein composition of different compartments and can thus constitute a starting point for further in-depth functional studies.

Next, we compared the ZC3 proteomics and transcriptomics data for each type of pan-cancer patients from the CPTAC to provide complementary information on the protein expression and gene expression profiles. Our results showed that the correlation between ZC3 protein expression and mRNA expression was moderate. This correlation varies among different cancer types, with the highest correlation observed in head and neck squamous cell carcinoma and the lowest correlation observed in glioblastoma. Particularly, the increase in ZC3 protein accumulation did not coincide with a corresponding increase in ZC3 mRNA expression in GBM, ccRCC and PDAC. The discrepancy between ZC3 proteomics and transcriptomics data may reflect the complex regulatory mechanisms that control post-transcriptional gene expression, including RNA stability, splicing, and translation efficiency. In addition, the protein expression levels of ZC3 may be influenced by post-translational modifications, such as ubiquitination and phosphorylation, which are not directly reflected in mRNA expression levels.

We further revealed the phosphorylation of ZC3 in three cancer types (ccRCC, GBM, and PDAC) which has low correlation between ZC3 mRNA expression and protein abundance. Our results showed that ZC3 is phosphorylated at several sites, including Ser-108, Ser-290, and Ser-313, in GBM, but not in others. Phosphorylation of ZC3 may affect its interaction with other proteins and RNAs that play a role in cancer progression. For example, ZC3 has been shown to interact with the mRNA decay machinery, including the exosome and decapping enzymes (Singh et al., 2014). Phosphorylation of ZC3 may affect its recruitment to these complexes and therefore alter mRNA stability and decay rates.

Genetic alterations, such as mutations and copy number variations, are common events in cancer that can affect the tumor biology behavior. ZC3 gene was altered in some cancer types, such as breast cancer and endometrial cancer, with a frequency ranging from 1 to 9%. The most frequent genetic alteration was amplification, followed by missense mutation, and deep deletion. The association between ZC3 genetic alteration and cancer progression remains unclear. However, our finding suggests that mutations in the ZC3 gene may play a role in tumor development and warrants further investigation.

The discrepancy in the correlation between ZC3 expression and patient survival may be due to the different roles of ZC3 in different cancer types. However, in most case, the higher expression of ZC3 was associated with worse overall survival. ZC3 may promote tumor growth and metastasis by facilitating RNA processing and/or interacting with splicing factors. The correlation between ZC3 expression and immune infiltration may reflect the role of ZC3 in regulating immune-related genes and pathways. For example, ZC3 has been shown to interact with several immune-related genes, including interleukin-6 and a large group of interferon-stimulated genes (Darweesh et al., 2022), which play a role in anti-tumor immune response.

ZC3 is a member of the zinc finger CCCH-type containing protein family, which includes several proteins that are involved in RNA processing, splicing, and decay. Our results also showed that the main function of ZC3-related proteins was annotated as the mRNA processing cluster. This cluster includes several functional annotation groups, such as RNA binding, mRNA splicing, mRNA transport, and mRNA decay pathways. This functional annotation suggests that ZC3-related proteins are vital in regulating RNA post-transcriptional gene expression and metabolism, which are critical processes in cancer development and progression.

In summary, the findings of this study offer novel insights into the relationship among virus infection, ZC3 and pan-cancer. We also highlight the importance of multi-omics analysis in cancer research, which involves the integration of transcriptomic and proteomic data to achieve a comprehensive understanding of ZC3 underlying cancer development and progression. Further investigations are required to clarify the molecular mechanisms that underlie the correlation between ZC3 and cancer in-depth and to explore its potential as a diagnostic and therapeutic target in cancer.

## Data availability statement

The original contributions presented in the study are included in the article/Supplementary material, further inquiries can be directed to the corresponding author.

## Author contributions

JL: Writing—original draft. MS: Writing—original draft, Data curation, Methodology. ZL: Writing—original draft, Software. FN: Formal analysis, Project administration, Writing—original draft. BW: Validation, Writing—review & editing. DQ: Investigation, Resources, Writing—original draft. MH: Writing—review & editing.

## Supplementary material

Supplementary Figure S1 The relationship between ZC3 expression and prognosis verified by Starbase and Kaplan-Meier plotter tool. **(A)** The correlation between ZC3 expression and patient overall survival by starbase. **(B)** The correlation between ZC3 expression and patient overall survival by Kaplan–Meier plotter tool.

Supplementary Table S1 Descriptive statistics of ZC3 mRNA expression in TCGA cancer and normal tissues.

Supplementary Table S2 Independent samples *t*-tests of ZC3 mRNA between tumor and normal tissues.

Supplementary Table S3 Descriptive statistics of ZC3 protein expression in TCGA cancer and normal tissues.

Supplementary Table S4 Survival analysis table of pan-cancer analysis for ZC3H11A from starbase.
