# Supplementary figures and images for: Analysis of the mRNA export protein ZC3H11A in HCMV infection and pan-cancer
Source: Front Microbiol. 2023 Nov 15;14:1296725. doi: 10.3389/fmicb.2023.1296725 (PMC10684726; doi:10.3389/fmicb.2023.1296725)

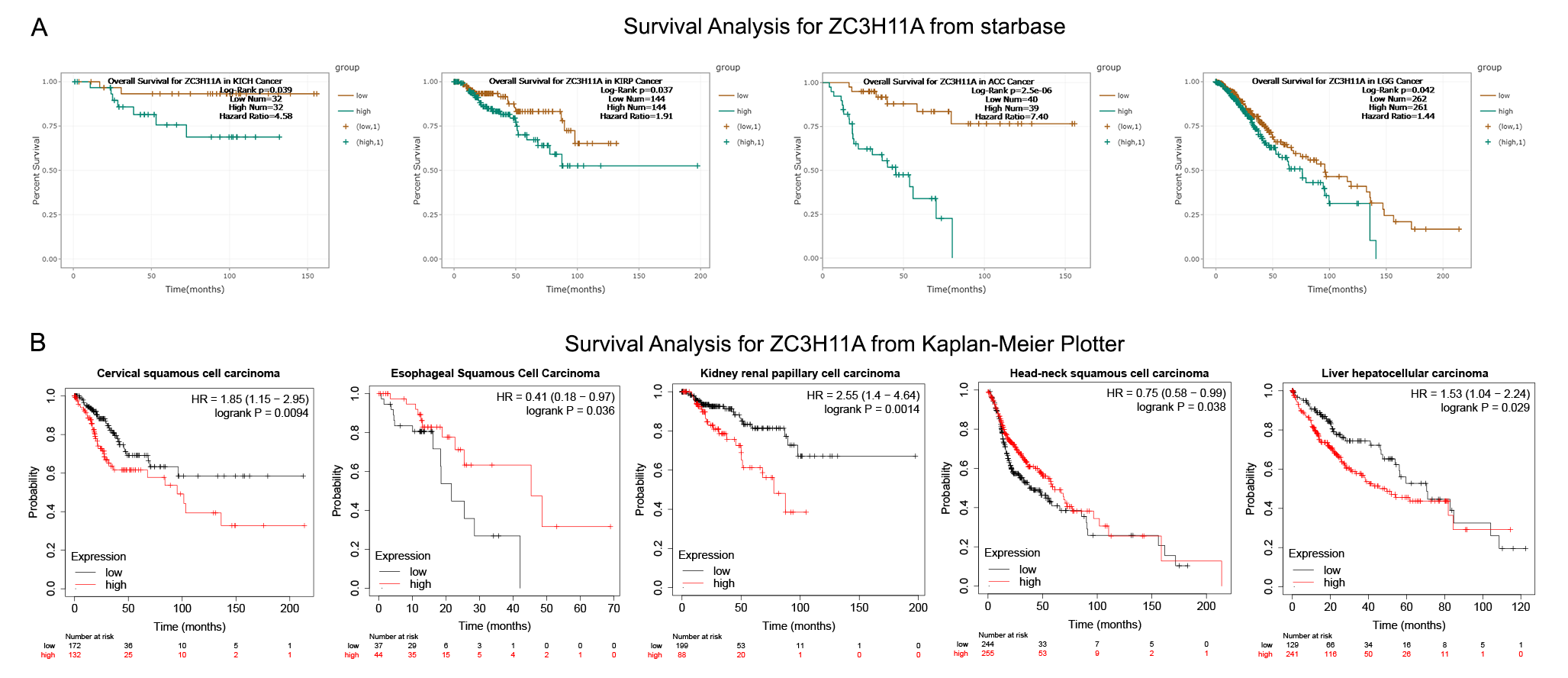

Supplement: Supplementary Figure S1 — The relationship between ZC3 expression and prognosis verified by Starbase and Kaplan-Meier plotter tool. (A) The correlation between ZC3 expression and patient overall survival by starbase. (B) The correlation between ZC3 expression and patient overall survival by Kaplan–Meier plotter tool. [file Image_1.TIF]
