# Supplementary material for: Analysis of the mRNA export protein ZC3H11A in HCMV infection and pan-cancer
Source: Front Microbiol. 2023 Nov 15;14:1296725. doi: 10.3389/fmicb.2023.1296725 (PMC10684726; doi:10.3389/fmicb.2023.1296725)
Supplement: Supplementary Table S1 — Descriptive statistics of ZC3 mRNA expression in TCGA cancer and normal tissues. [file Table_1.docx]

**Table S1. Descriptive statistics of ZC3 mRNA expression in TCGA cancer and normal tissues**

| **TCGA (Abbr.)** | **Group** | **Numbers** | **Median** | **IQR** | **Mean** | **SD** | **SE** |
| --- | --- | --- | --- | --- | --- | --- | --- |
| BLCA | Normal | 19 | 5.206 | 0.397 | 5.046 | 0.449 | 0.103 |
|  | Tumor | 414 | 5.145 | 0.758 | 5.092 | 0.598 | 0.029 |
| BRCA | Normal | 113 | 5.935 | 0.521 | 5.896 | 0.458 | 0.043 |
|  | Tumor | 1109 | 6.008 | 0.819 | 5.981 | 0.673 | 0.02 |
| CESC | Normal | 3 | 5.528 | 0.482 | 5.429 | 0.489 | 0.283 |
|  | Tumor | 306 | 5.503 | 0.715 | 5.442 | 0.589 | 0.034 |
| CHOL | Normal | 9 | 3.924 | 0.355 | 3.938 | 0.266 | 0.089 |
|  | Tumor | 36 | 5.644 | 1.106 | 5.796 | 0.743 | 0.124 |
| COAD | Normal | 41 | 4.605 | 0.367 | 4.587 | 0.27 | 0.042 |
|  | Tumor | 480 | 4.973 | 0.783 | 4.89 | 0.664 | 0.03 |
| ESCA | Normal | 11 | 5.111 | 0.649 | 5.199 | 0.661 | 0.199 |
|  | Tumor | 162 | 5.855 | 0.626 | 5.863 | 0.523 | 0.041 |
| GBM | Normal | 5 | 4.716 | 0.16 | 4.828 | 0.342 | 0.153 |
|  | Tumor | 169 | 5.017 | 0.645 | 5.046 | 0.785 | 0.06 |
| HNSC | Normal | 44 | 4.675 | 0.699 | 4.56 | 0.558 | 0.084 |
|  | Tumor | 502 | 5.333 | 0.73 | 5.241 | 0.631 | 0.028 |
| KICH | Normal | 24 | 5.33 | 0.53 | 5.31 | 0.433 | 0.088 |
|  | Tumor | 65 | 4.084 | 0.879 | 4.045 | 0.663 | 0.082 |
| KIRC | Normal | 72 | 5.051 | 0.627 | 5.035 | 0.485 | 0.057 |
|  | Tumor | 539 | 5.175 | 0.837 | 5.08 | 0.674 | 0.029 |
| KIRP | Normal | 32 | 4.814 | 0.519 | 4.924 | 0.425 | 0.075 |
|  | Tumor | 289 | 4.865 | 0.982 | 4.777 | 0.756 | 0.044 |
| LIHC | Normal | 50 | 3.638 | 0.577 | 3.611 | 0.439 | 0.062 |
|  | Tumor | 374 | 4.432 | 1.024 | 4.45 | 0.79 | 0.041 |
| LUAD | Normal | 59 | 5.201 | 0.525 | 5.061 | 0.488 | 0.064 |
|  | Tumor | 535 | 5.559 | 0.765 | 5.508 | 0.64 | 0.028 |
| LUSC | Normal | 49 | 5.136 | 0.648 | 5.117 | 0.402 | 0.057 |
|  | Tumor | 502 | 5.559 | 0.686 | 5.52 | 0.595 | 0.027 |
| PAAD | Normal | 4 | 5.367 | 0.444 | 5.492 | 0.481 | 0.241 |
|  | Tumor | 178 | 5.473 | 0.531 | 5.426 | 0.543 | 0.041 |
| PCPG | Normal | 3 | 4.763 | 0.502 | 5.051 | 0.561 | 0.324 |
|  | Tumor | 183 | 5.172 | 0.722 | 5.079 | 0.582 | 0.043 |
| PRAD | Normal | 52 | 5.367 | 0.707 | 5.324 | 0.469 | 0.065 |
|  | Tumor | 499 | 5.418 | 0.7 | 5.338 | 0.58 | 0.026 |
| READ | Normal | 10 | 4.774 | 0.3 | 4.735 | 0.412 | 0.13 |
|  | Tumor | 167 | 4.913 | 0.791 | 4.839 | 0.706 | 0.055 |
| SARC | Normal | 2 | 4.231 | 0.265 | 4.231 | 0.374 | 0.265 |
|  | Tumor | 263 | 5.337 | 0.825 | 5.326 | 0.725 | 0.045 |
| SKCM | Normal | 1 | 5.581 | 0 | 5.581 |  |  |
|  | Tumor | 471 | 5.638 | 0.994 | 5.508 | 0.843 | 0.039 |
| STAD | Normal | 32 | 4.46 | 0.945 | 4.281 | 0.751 | 0.133 |
|  | Tumor | 375 | 5.303 | 0.937 | 5.274 | 0.642 | 0.033 |
| THCA | Normal | 58 | 5.772 | 0.341 | 5.651 | 0.483 | 0.063 |
|  | Tumor | 510 | 5.442 | 0.721 | 5.329 | 0.57 | 0.025 |
| THYM | Normal | 2 | 6.209 | 0.227 | 6.209 | 0.32 | 0.227 |
|  | Tumor | 119 | 5.722 | 0.839 | 5.6 | 0.748 | 0.069 |
| UCEC | Normal | 35 | 5.623 | 0.452 | 5.581 | 0.309 | 0.052 |
|  | Tumor | 552 | 5.138 | 0.988 | 5.129 | 0.771 | 0.033 |

**Table S2. Independent samples t-tests of ZC3 mRNA between tumor and normal tissues**

| **TCGA (Abbr.)** | **Group1 [Numbers]** | **Group2 [Numbers]** | **t value** | **df** | **Group2 v.s. Group1** | **95%CI** | **p value** |
| --- | --- | --- | --- | --- | --- | --- | --- |
| CHOL | Normal [9] | Tumor [36] | 7.328 | 43 | 1.858 | 1.347 - 2.369 | 0 |
| STAD | Normal [32] | Tumor [375] | 8.276 | 405 | 0.992 | 0.757 - 1.228 | 0 |
| LIHC | Normal [50] | Tumor [374] | 7.353 | 422 | 0.839 | 0.615 - 1.064 | 0 |
| HNSC | Normal [44] | Tumor [502] | 6.925 | 544 | 0.681 | 0.488 - 0.874 | 0 |
| ESCA | Normal [11] | Tumor [162] | 4.008 | 171 | 0.664 | 0.337 - 0.991 | 0 |
| LUAD | Normal [59] | Tumor [535] | 5.199 | 592 | 0.447 | 0.278 - 0.616 | 0 |
| LUSC | Normal [49] | Tumor [502] | 4.643 | 549 | 0.403 | 0.233 - 0.574 | 0 |
| COAD | Normal [41] | Tumor [480] | 2.901 | 519 | 0.303 | 0.098 - 0.508 | 0.004 |
| GBM | Normal [5] | Tumor [169] | 0.619 | 172 | 0.218 | -0.478 - 0.915 | 0.537 |
| READ | Normal [10] | Tumor [167] | 0.457 | 175 | 0.103 | -0.342 - 0.549 | 0.648 |
| BRCA | Normal [113] | Tumor [1109] | 1.319 | 1220 | 0.085 | -0.042 - 0.213 | 0.188 |
| BLCA | Normal [19] | Tumor [414] | 0.331 | 431 | 0.046 | -0.227 - 0.319 | 0.741 |
| KIRC | Normal [72] | Tumor [539] | 0.546 | 609 | 0.045 | -0.116 - 0.206 | 0.586 |
| PCPG | Normal [3] | Tumor [183] | 0.083 | 184 | 0.028 | -0.64 - 0.696 | 0.934 |
| PRAD | Normal [52] | Tumor [499] | 0.165 | 549 | 0.014 | -0.15 - 0.177 | 0.869 |
| CESC | Normal [3] | Tumor [306] | 0.039 | 307 | 0.013 | -0.658 - 0.685 | 0.969 |
| PAAD | Normal [4] | Tumor [178] | -0.238 | 180 | -0.065 | -0.606 - 0.476 | 0.812 |
| KIRP | Normal [32] | Tumor [289] | -1.077 | 319 | -0.146 | -0.414 - 0.121 | 0.282 |
| THCA | Normal [58] | Tumor [510] | -4.142 | 566 | -0.322 | -0.475 - -0.17 | 0 |
| UCEC | Normal [35] | Tumor [552] | -3.454 | 585 | -0.453 | -0.71 - -0.195 | 0.001 |
| KICH | Normal [24] | Tumor [65] | -8.673 | 87 | -1.265 | -1.555- -0.975 | 0 |

**Table S3. Descriptive statistics of ZC3 protein expression in TCGA cancer and normal tissues**

| **Cancer Type** | **low** | **q1** | **median** | **q3** | **high** | **Statistical significance (Normal-vs-Tumor)** |
| --- | --- | --- | --- | --- | --- | --- |
| Breast cancer - Normal (n=18) | -1.921 | -1.182 | -0.971 | -0.744 | -0.362 | 2.65E-11 |
| Breast cancer - Tumor (n=125) | -1.91 | -0.613 | -0.109 | 0.523 | 2.24 |  |
| Colon cancer - Normal (n=100) | -4.386 | -2.702 | -1.934 | -1.498 | 0.107 | 7.66E-29 |
| Colon cancer - Tumor (n=97) | -2.795 | -0.82 | 0.017 | 0.525 | 2.49 |  |
| Ovarian cancer - Normal (n=25) | -2.612 | -1.673 | -1.237 | -0.953 | -0.192 | 4.23E-08 |
| Ovarian cancer - Tumor (n=100) | -1.762 | -0.445 | 0.019 | 0.556 | 1.898 |  |
| Clear cell renal cell carcinoma - Normal (n=84) | -1.952 | -0.808 | -0.178 | 0.408 | 2.017 | 1.07E-01 |
| Clear cell renal cell carcinoma - Tumor (n=110) | -2.232 | -0.571 | 0 | 0.82 | 2.411 |  |
| Uterine corpus endometrial carcinoma - Normal (n=31) | -1.925 | -1.447 | -1.313 | -1.005 | -0.544 | 2.43E-15 |
| Uterine corpus endometrial carcinoma - Tumor (n=100) | -2.315 | -0.953 | 0 | 0.652 | 1.83 |  |
| Lung adenocarcinoma - Normal (n=111) | -2.957 | -1.909 | -1.576 | -1.247 | -0.333 | 6.42E-33 |
| Lung adenocarcinoma - Tumor (n=111) | -1.872 | -0.592 | 0.002 | 0.819 | 2.746 |  |
| Pancreatic adenocarcinoma - Normal (n=74) | -2.658 | -0.842 | -0.266 | 0.408 | 1.16 | 4.83E-02 |
| Pancreatic adenocarcinoma - Tumor (n=137) | -2.364 | -0.632 | -0.022 | 0.616 | 2.261 |  |
| Head and neck squamous carcinoma - Normal (n=71) | -1.779 | -0.877 | -0.393 | 0.016 | 1.422 | 4.22E-04 |
| Head and neck squamous carcinoma - Tumor (n=108) | -1.809 | -0.642 | -0.019 | 0.58 | 2.405 |  |
| Glioblastoma multiforme - Normal (n=10) | -1.377 | -1.004 | -0.866 | -0.697 | -0.425 | 2.10E-08 |
| Glioblastoma multiforme - Tumor (n=99) | -1.809 | -0.553 | -0.069 | 0.391 | 1.8 |  |
| Hepatocellular carcinoma - Normal (n=165) | -2.266 | -1.001 | -0.532 | -0.029 | 1.169 | 2.65E-09 |
| Hepatocellular carcinoma - Tumor (n=165) | -2.077 | -0.605 | -0.005 | 0.826 | 2.234 |  |

**Table S4. Survival Analysis Table of Pan-Cancer Analysis for ZC3H11A from starbase**

| **Cancer** | **Cancer Full Name** | **CancerNum** | **Median** | **coef** | **HR** | **pValue** |
| --- | --- | --- | --- | --- | --- | --- |
| ACC | Adrenocortical Carcinoma | 79 | 9.27 | 2 | 7.4 | 0.0000025 |
| KIRP | Kidney Renal Papillary Cell Carcinoma | 288 | 10.7 | 0.65 | 1.91 | 0.037 |
| KICH | Kidney Chromophobe | 64 | 6.73 | 1.52 | 4.58 | 0.039 |
| LGG | Brain Lower Grade Glioma | 523 | 10.76 | 0.36 | 1.44 | 0.042 |
| THCA | Thyroid Carcinoma | 509 | 12.82 | 0.96 | 2.62 | 0.064 |
| PRAD | Prostate Adenocarcinoma | 495 | 13.21 | 1.19 | 3.28 | 0.073 |
| HNSC | Head and Neck Squamous Cell Carcinoma | 495 | 12.42 | -0.24 | 0.78 | 0.077 |
| CESC | Cervical Squamous Cell Carcinoma and Endocervical Adenocarcinoma | 306 | 14.62 | 0.39 | 1.48 | 0.096 |
| UVM | Uveal Melanoma | 80 | 7.48 | 0.67 | 1.95 | 0.14 |
| PCPG | Pheochromocytoma and Paraganglioma | 183 | 11.91 | 1.1 | 3.02 | 0.16 |
| MESO | Mesothelioma | 85 | 13.12 | 0.32 | 1.38 | 0.18 |
| DLBC | Lymphoid Neoplasm Diffuse Large B-cell Lymphoma | 47 | 11.66 | 0.93 | 2.55 | 0.19 |
| OV | Ovarian Serous Cystadenocarcinoma | 374 | 10.82 | 0.11 | 1.12 | 0.4 |
| ESCA | Esophageal Carcinoma | 162 | 18.48 | -0.21 | 0.81 | 0.41 |
| TGCT | Testicular Germ Cell Tumors | 139 | 12.45 | -0.86 | 0.42 | 0.45 |
| PAAD | Pancreatic Adenocarcinoma | 178 | 14.24 | 0.16 | 1.17 | 0.46 |
| READ | Rectum Adenocarcinoma | 159 | 9.93 | -0.3 | 0.74 | 0.46 |
| SKCM | Skin Cutaneous Melanoma | 440 | 15.18 | -0.1 | 0.9 | 0.46 |
| KIRC | Kidney Renal Clear Cell Carcinoma | 517 | 11.79 | -0.1 | 0.9 | 0.51 |
| LUAD | Lung Adenocarcinoma | 503 | 14.96 | 0.09 | 1.09 | 0.55 |
| BRCA | Breast Invasive Carcinoma | 1082 | 19.06 | 0.09 | 1.1 | 0.58 |
| SARC | Sarcoma | 261 | 10.89 | -0.11 | 0.9 | 0.59 |
| UCS | Uterine Carcinosarcoma | 56 | 14.37 | -0.19 | 0.83 | 0.59 |
| STAD | Stomach Adenocarcinoma | 365 | 14.52 | -0.08 | 0.92 | 0.62 |
| THYM | Thymoma | 118 | 15.89 | -0.25 | 0.78 | 0.71 |
| COAD | Colon Adenocarcinoma | 447 | 9.8 | -0.07 | 0.94 | 0.74 |
| LIHC | Liver Hepatocellular Carcinoma | 369 | 7.94 | 0.05 | 1.05 | 0.78 |
| CHOL | Cholangiocarcinoma | 36 | 16.68 | 0.13 | 1.14 | 0.79 |
| BLCA | Bladder Urothelial Carcinoma | 406 | 11.62 | -0.04 | 0.96 | 0.8 |
| LAML | Acute Myeloid Leukemia | 75 | 24.65 | -0.02 | 0.98 | 0.93 |
| LUSC | Lung Squamous Cell Carcinoma | 469 | 14.56 | -0.01 | 0.99 | 0.93 |
| [UCEC](https://starbase.sysu.edu.cn/panGeneSurvivalExp.php) | Uterine Corpus Endometrial Carcinoma | 537 | 12.16 | 0.01 | 1.01 | 0.95 |
