# Supplementary material for: Analysis of the mRNA export protein ZC3H11A in HCMV infection and pan-cancer
Source: Front Microbiol. 2023 Nov 15;14:1296725. doi: 10.3389/fmicb.2023.1296725 (PMC10684726; doi:10.3389/fmicb.2023.1296725)
Supplement: Supplementary file 3 [file Data_Sheet_1.docx]

cptac.download(dataset="colon", version="latest")

co = cptac.Colon()

colon_cross = co.join_omics_to_omics(df1_name="proteomics", df2_name="transcriptomics",

genes1="ZC3H11A", genes2="ZC3H11A")

sns.set(style="darkgrid")

plot = sns.regplot(x=colon_cross.columns[0], y=colon_cross.columns[1], data=colon_cross)

plot.set(xlabel='Proteomics', ylabel='Transcriptomics', title='Proteomics vs. Transcriptomics for the ZC3H11A gene')

plt.show()
